# Fast Ultrasensitive Sensing Strip for the Electrochemical Determination of Vitamin B6

**DOI:** 10.3390/biology15141098

**Published:** 2026-07-08

**Authors:** Ergün Yukmel Rasit, Raluca-Ioana Stefan-van Staden, Damaris-Cristina Gheorghe

**Affiliations:** 1 Faculty of Chemical Engineering and Biotechnologies, National University of Science and Technology Politehnica of Bucharest, 011061 Bucharest, Romania; 2 Laboratory of Electrochemistry and PATLAB, National Institute of Research for Electrochemistry and Condensed Matter, 060021 Bucharest, Romania

**Keywords:** vitamin B6, sensing strip, cyclodextrin, pharmaceuticals, food

## Abstract

Vitamin B6 is a coenzyme involved in more than 150 biochemical reactions in the body. Its intake has favorable effects for many diseases like Parkinson’s disease, glaucoma, and endometriosis. Vitamin B6 can be obtained from tablets, aqua vitamin, and a variety of food sources, including meat, fish, and vegetables. There is a need for process analytical control for fast, ultrasensitive, and selective methods of determination of vitamin B6 in order to produce drugs of high pharmaceutical purity. Also, the on-site determination of vitamin B6 in vegetables can contribute to the fast, real-time control of the quality of vegetables. Therefore, a fast ultrasensitive sensing strip based on the immobilization of heptakis(2,3,6-tri-O-methyl)-beta-cyclodextrin on a matrix containing single-walled carbon nanotubes was constructed and used for on-site pharmaceutical process control of vitamin B6, as well as for on-site quality control of food. The sensing strip provided two linear concentration ranges: the first one between 1.69 × 10^−15^ and 1.69 × 10^−5^ g mL^−1^ with a sensitivity of 6.96 × 10^4^ s^−1^ g^−1^ mL, and the second one between 1.69 × 10^−5^ and 1.69 × 10^−3^ g mL^−1^ with a sensitivity of 3.65 × 10^2^ s^−1^ g^−1^ mL. The limit of quantification was 1.69 fg mL^−1^. A % recovery higher than 94.50% was determined for Vitamin B6 in tablets, aqua vitamin, and avocado, with % relative standard deviations lower than 1.00%. A lower determination limit was obtained when the sensing strip was used for the assay of vitamin B6 in different samples compared with other methods used to date for its assay. Also, the results of the analysis were obtained faster, in real time, compared with other methods for which a long sample processing time is needed before analysis.

## Simple Summary

More than 150 biochemical reactions involve vitamin B6 as a coenzyme. Parkinson’s disease, glaucoma, and endometriosis improve with its use. Tablets, aqua vitamin, and avocados contain vitamin B6. To create high-purity pharmaceuticals, process analytical control requires quick, ultrasensitive, and selective vitamin B6 determination methods. Additionally, on-site B6 vitamin determination in vegetables can help control vegetable quality in real time. Thus, a fast, ultra-sensitive sensing strip was developed, which can be used for the quality control of vitamin B6 from various types of samples (pharmaceutical compounds containing vitamin B6, water vitamins, avocado, and surface water).

## 1. Introduction

Vitamin B6 (pyridoxine) is a water-soluble vitamin essential for brain function, immune support, metabolism, and red blood cell formation [1]. Vitamin B6 plays the role of a coenzyme, being involved in more than 150 biochemical reactions in the body [1]. Vitamin B6 influences sleep, mood, and cognition by helping with the production of red blood cells and can prevent anemia [1]. It can also reduce nausea in pregnancy, helping fetal brain development [1]. In the immune system, vitamin B6 helps with the production of antibodies and lymphocytes [1]. A daily intake of 1.3 mg of vitamin B6 is recommended [1]. Vitamin B6 can be taken from pharmaceutical tablets, different beverages, and vegetables like avocado. A positive correlation between the endometriosis risk and vitamin B6 intake was found by Yin et al. [2], while Zhang et al. [3] demonstrated that a higher intake of vitamin B6 before Parkinson’s disease may decrease the risk of death in individuals with this disease. A positive correlation between the intake of vitamin B6 and prevention of glaucoma was also observed [4].

Different methods of analysis were proposed for the determination of vitamin B6. These methods include: chromatographic methods of analysis [5,6], fluorescence methods of analysis [7], ultraviolet spectrophotometric method [8], quantum dots-based analysis [9], and electroanalysis using different types of sensors [9,10,11,12,13,14]. The literature survey demonstrated that electrochemical sensors played an important role in the reliable determination of vitamin B6 [9,10,11,12,13,14]. The evolution of electrochemical sensors’ technology in the later period of time may even increase the reliability of such determinations [15,16,17,18].

Stochastic sensors constitute an emerging category of electrochemical sensors that utilize molecular channels or pores integrated into conductive materials, including carbon-based nanomaterials, graphene, or metallic composites. Their operation depends on quantifying current variations under a fixed potential. Upon the entry of a molecule into the channel, it momentarily obstructs ion transport, producing a distinctive t_off_ value that functions as a signature for qualitative identification. The molecule’s subsequent interaction within the channel generates a t_on_ value that is inversely proportionate to its concentration, facilitating quantitative determination for molecules at low concentrations in complex matrices [19]. These types of sensors offer several advantages over traditional sensors, particularly regarding their enhanced sensitivity, adaptability, cost-effectiveness, and capacity for real-time monitoring. Furthermore, the analyzed samples necessitate no pretreatment prior to analysis. Their response is independent of the matrix’s components, depending only on the size (length, volume), geometry, and stereochemistry of the molecule that is analyzed. Stochastic strips were introduced as a subclass of the stochastic sensors earlier and are reliable disposable sensors used for the on-site, fast, real-time analysis of compounds of interest.

This paper proposes a new design for a sensing strip, based on the immobilization of the heptakis(2,3,6-tri-O-methyl)-beta-cyclodextrin on a matrix containing single-walled carbon nanotubes. The single-walled carbon nanotubes (SWCNT) were deposited on a ceramic material according to the procedure proposed by Rezaree et al. [20]; also, to enhance the electrical conductivity, the SWCNT layer was washed with Brønsted acid (mixture of HCl, HNO_3_, and H_2_SO_4_). SWCNT was chosen as the supporting matrix due to its superior electrical conductivity, extensive surface area, and exceptional electrochemical characteristics, which improve signal quality and sensitivity. SWCNT provides a stable layer for the channels of the modifier: heptakis(2,3,6-tri-O-methyl)-β-cyclodextrin. The β-cyclodextrin side of the modifier molecule has a cavity that can function as a nanochannel needed for the stochastic signal development. The channels are needed for stochastic sensing because the stochastic sensing is based on the channel conductivity. Measurements done before the modification of the strip’s surface with heptakis(2,3,6-tri-O-methyl)-β-cyclodextrin showed no current development/no stochastic signal; after the addition of the modifier, a well-defined stochastic signal was obtained. The current development in stochastic sensing is based on two steps.

**Scheme 1:**
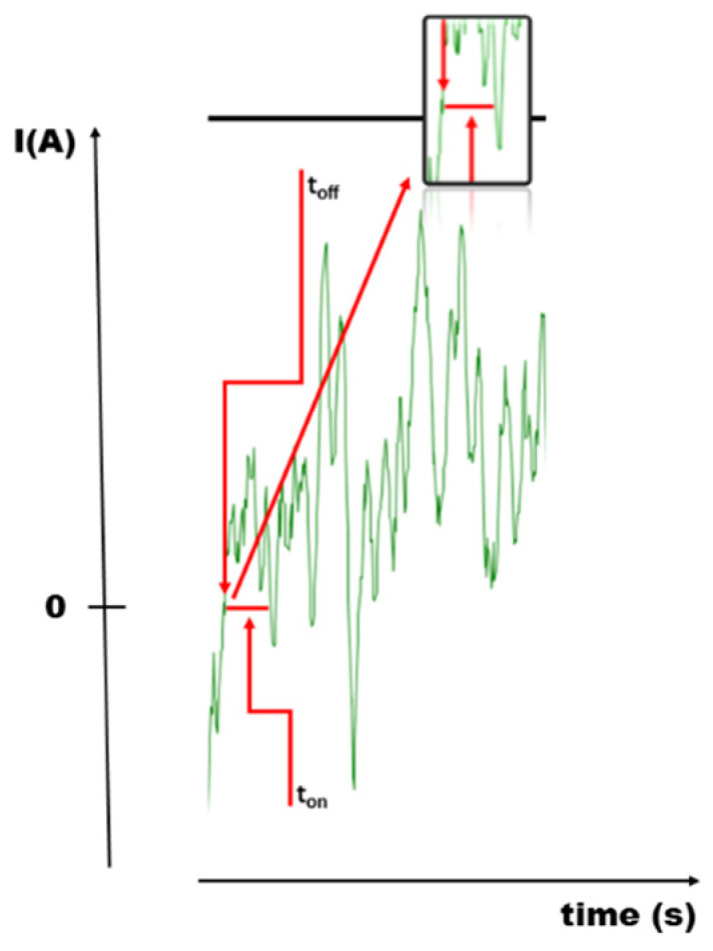
Current development in stochastic sensing.

The molecular recognition step is associated with the entrance of vitamin B6 into the channel (Scheme 1), the current cannot pass through the channel, and a 0 A value is recorded until the molecule is getting inside the channel; the step is associated with the t_off_ value read on the diagram, and because this value is specific to the analyte determined, it is called the signature of vitamin B6. The second step takes place inside the channel, when binding to the wall of the channel and redox processes are taking place; this step is characterized by the t_on_ value in the diagram (Scheme 1). The higher the concentration of vitamin B6, the lower the value of t_on_. After this step, the molecule is getting out, and the next molecule from the solution is entering the channel.

## 2. Materials and Methods

### 2.1. Reagents and Materials

Vitamins B1 (thiamine), B2 (riboflavin), B3 (niacin), B5 (pantothenic acid), B6, B7 (biotin), B9 (folic acid), B12, C, and E, SWCNT powder, MgCl_2_, HCl, HNO_3_, and H_2_SO_4_ of analytical purity were purchased from Sigma-Aldrich (St. Louis, MO, USA). Heptakis(2,3,6-tri-O-methyl)-beta-cyclodextrin (TRIMEB) was provided by Carbohyde (Budapest, Hungary).

A series of vitamin B6 solutions (concentrations ranging from 1.69 × 10^−19^ to 1.69 × 10^−2^ g mL^−1^) were obtained using the serial dilution method; all solutions were buffered with a phosphate-buffered solution (pH = 6.74). Class 1 accuracy measuring glassware was used at all stages of solution preparation and deposition.

### 2.2. Apparatus

The stochastic measurements were performed using an EmStat Pico mini potentiostat (PalmSens BV, Houten, The Netherlands) connected to a smartphone operating the PStouch mobile application version 2.7.

### 2.3. The Design of the Sensing Strip Used for the Determination of Vitamin B6

**Figure 1:**
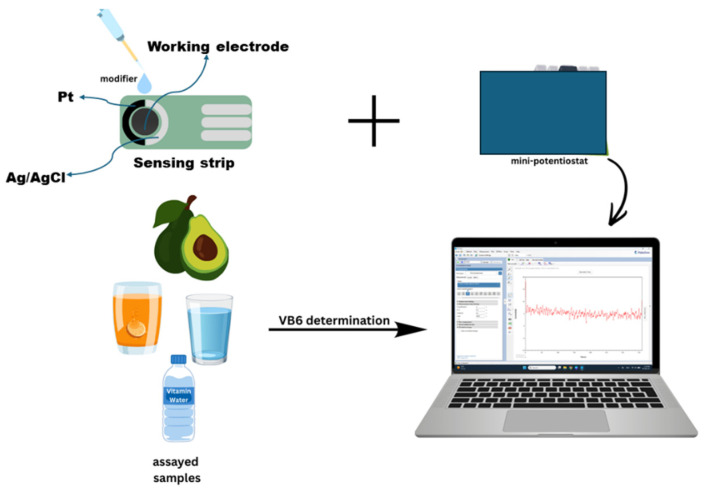
Design of sensing strips and the setup for analysis of vitamin B6.

First, the working electrode, with a thickness of 2 mm and a diameter of 0.4 cm, was deposited on a ceramic material using the method proposed by Rezaree et al. [20]. The floating catalyst chemical vapor deposition (FCCVD) method was used, with a flow rate of 200 sccm of mixed gas (Ar and H_2_) (Figure 1). The surface was treated with Brønsted acid (mixture of HCl, HNO_3_, and H_2_SO_4_) in order to enhance the electrical properties of the SWCNT. The Pt (auxiliary electrode) and the Ag/AgCl (pseudoreference electrode) wires were drawn as two semicircles around the working electrode, using cold plasma, after covering the working electrode with a special plastic mask (Figure 1). For the Ag/AgCl pseudoreference electrode, first the Ag was deposited, followed by the deposition of AgCl. For their deposition, the following parameters were applied: the chamber pressure was maintained at 2 × 10^−5^ mBar, while the electrical deposition parameters for initiating a single plasma plume from the material comprised a filament current of 75 A, a plasma current of 1.8 A, a plasma voltage of 800 V, and a deposition period of 10 min.

The working electrode was then modified with a solution of heptakis(2,3,6-tri-O-methyl)-beta-cyclodextrin (1 × 10^−3^ mol L^−1^), by placing on the whole surface of the working electrode, of 5 µL of this solution. The solution was left to dry on top of the working electrode for 4 h at 25 °C; during this time, the sensor was kept inside a glass recipient.

### 2.4. Stochastic Method

A chronoamperometric technique was used for all measurements. A constant potential of 400 mV vs. Ag/AgCl was applied for all measurements. Application of a higher potential gave t_off_ values of ns, which could not be reliably read even using the automatic reading system. Applying a lower potential than 400 mV does not facilitate the extraction of vitamin B6 into the channel; no stochastic signal was obtained.

Also, before the modification with the TRIMEB, the sensing strip was checked for any stochastic response.

The sensing strip was introduced into each standard solution from the series of vitamin B6 prepared (concentrations ranging from 1.69 × 10^−19^ to 1.69 × 10^−2^ g mL^−1^). The linear regression method was used to obtain the a and b parameters of the equation of calibration: 1/t_on_ = a + b × C_vitamin B6_, which correlates the t_on_ values with the concentrations of vitamin B6. By introducing the t_on_ value into the equation of calibration, it is possible to determine the concentration of vitamin B6.

For data processing and initial selection of the signature specific for vitamin B6, the following steps were taken: first, the buffer solution (phosphate buffer pH = 6.74) was scanned and all values of t_off_—signatures specific for buffer components were measured. To this buffer solution, from the standard solution containing vitamin B6 and buffer solution was added a volume to give a concentration of 1.69 × 10^−19^ g mL^−1^; three measurements were done for this solution, and the signatures obtained for the buffer solution and the solution containing the vitamin B6 were compared when a new value of 0.4 s was identified; this value was attributed to vitamin B6 (Figure S2 from Supplementary Materials). It was also observed that from the concentration of 1.69 × 10^−15^ to 1.69 × 10^−5^ g mL^−1^, there is a direct proportionality between the 1/t_on_ values and concentrations of vitamin B6; the t_on_ values decreased with increasing concentration. A different sensitivity and proportionality were statistically determined in the range 1.69 × 10^−5^–1.69 × 10^−3^ g mL^−1^. All statistical calculations, including the parameters of the equations of calibration, were performed using the Vassar Stats website for statistical computation (http://vassarstats.net). Formulas used for calculation of parameters a, b, and r of the equations of calibration are also shown in the Supplementary Materials section.

### 2.5. Samples

One surface water sample, Aqua Vitamin MG with a content of 0.21 mg vitamin B6 per L (bought from supermarket), two types of soluble tablets containing vitamin B6, one containing only vitamin B6 (5 mg per tablet), and MgCl_2_, and the other one containing 1.40 mg of vitamin B6 and vitamins B1, B2, B3, B5, B7, B12, C, and E. Avocado was also analyzed for its content in vitamin B6. For its analysis, the avocado was peeled, and its pulp was analyzed as is by placing it on the active surface of the sensor. The surface water samples, aqua Vitamin MG, and avocado were analyzed without any sampling. Ten soluble tablets of each kind were analyzed; each tablet was dissolved in 10 mL of a mixture of distilled water: buffer = 1:1 (*v*/*v*).

## 3. Results and Discussion

### 3.1. Response Characteristics of the Sensing Strip

The response characteristics of the sensing strip are shown in Table 1.

Two linear concentration ranges were recorded (graphs shown; some of the diagrams contributing to results in Table 1 are shown in Figure S2). The first one is on a very wide range 1.69 × 10^−15^–1.69 × 10^−5^ g mL^−1^, and with the highest sensitivity: 6.96 × 10^4^ s^−1^ g^−1^ mL. The second one is immediately following on two decades of concentration (1.69 × 10^−5^–1.69 × 10^−3^ g mL^−1^) with a smaller sensitivity than the first one, but still high: 3.65 × 10^2^ s^−1^ g^−1^ mL. The limit of determination (determined accordingly with the IUPAC guidelines as the lowest concentration found on the linear concentration range [21]) is very low, of 1.69 fg mL^−1^. While the second linear concentration range can be applied for the determination of vitamin B6 in pharmaceutical products and in water containing vitamin B6, as their concentration is within that range, the first linear concentration range can be applied for the determination of vitamin B6 in surface waters.

The signature of vitamin B6 is 0.4 s, and it is used for identifying the signal given by vitamin B6 on the diagrams.

The 1/t_on_ parameter denotes the rate of successful molecule interaction events per unit time within the channel. As the vitamin B6 concentrations increase, a greater number of molecules enter the channel, resulting in an augmented frequency of interaction events and therefore an increase in 1/t_on_. Consequently, 1/t_on_ is directly proportional to molecule concentration and indicates the molecule-channel interactions in the stochastic sensing process.

A comparison between the limits of determination obtained using the ultrasensitive sensing strip and the limits of quantification for vitamin B6 obtained using the latest methods proposed for vitamin B6 determination is shown in Table 2.

The results from Table 2 show that the lowest limit of determination for the determination of vitamin B6 was obtained using the proposed ultrasensitive sensing strip.

Design reliability studies were performed by constructing ten sensing strips in accordance with the proposed design. Each of the strips was calibrated on both linear concentration ranges, and the relative standard deviations from the mean values were calculated: 0.12% was the value found for the wider linear concentration range, and for the other one, 0.11%. The values demonstrated the reliability of the design of the sensing strip.

The stability of the sensing strip was evaluated by testing it every day for a period of 30 days, and comparing the sensitivities obtained every day. Relative standard deviations from the mean of 0.21% and 0.20% were recorded for the wider and the smallest concentration ranges, proving good stability in time of the sensing strip (Figure S1).

### 3.2. Selectivity of the Ultrasensitive Sensing Strip

The high selectivity of the stochastic sensor for vitamin B6 is determined by the molecular recognition characteristics of the modified channel. Selectivity results only from variations in the capacity of molecules to enter and interact with the enclosed cyclodextrin cavity, resulting in unique signatures (t_off_ values). The selectivity of the proposed sensing strip is also given by the difference between the signature of vitamin B6 and the signatures of tested substances (as supposed interferences); for a difference of a minimum of 0.2 s, no interference is occurring. The interferences tested were selected from the compounds that may be found in the pharmaceutical compounds and in the surface water samples. Therefore, the following compounds were selected: Mg^2+^, vitamins B1, B3, B5, B7, B9, B12, C, E, amoxicillin, flurbiprofen. The signatures obtained are shown in Table 3. These compounds do not produce overlapping signatures due to variations in size, polarity, charge, and molecular conformation, which restrict their effective interaction within the channel.

As all signatures recorded for Mg^2+^, vitamins B1, B3, B5, B7, B9, B12, C, E, amoxicillin, and flurbiprofen are higher than 0.2 s than the one recorded for vitamin B6 (0.4 s), the proposed sensing strip is selective.

### 3.3. Validation of the Ultrasensitive Sensing Strip for Determination of Vitamin B6 from Tablets, Vitamin Water, and Surface Water Samples

**Figure 2:**
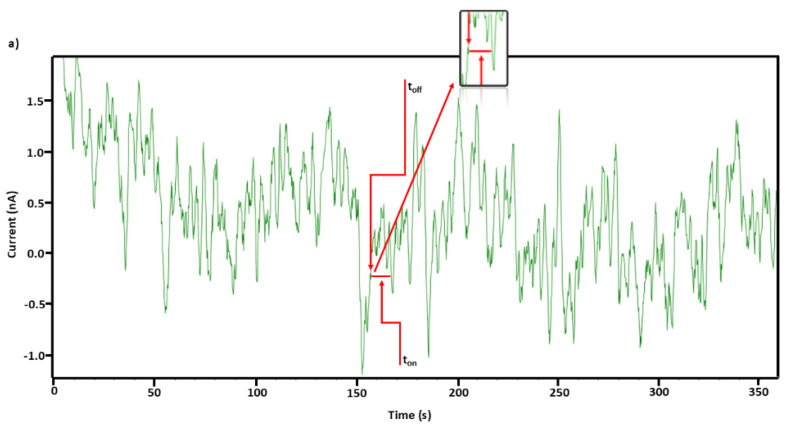

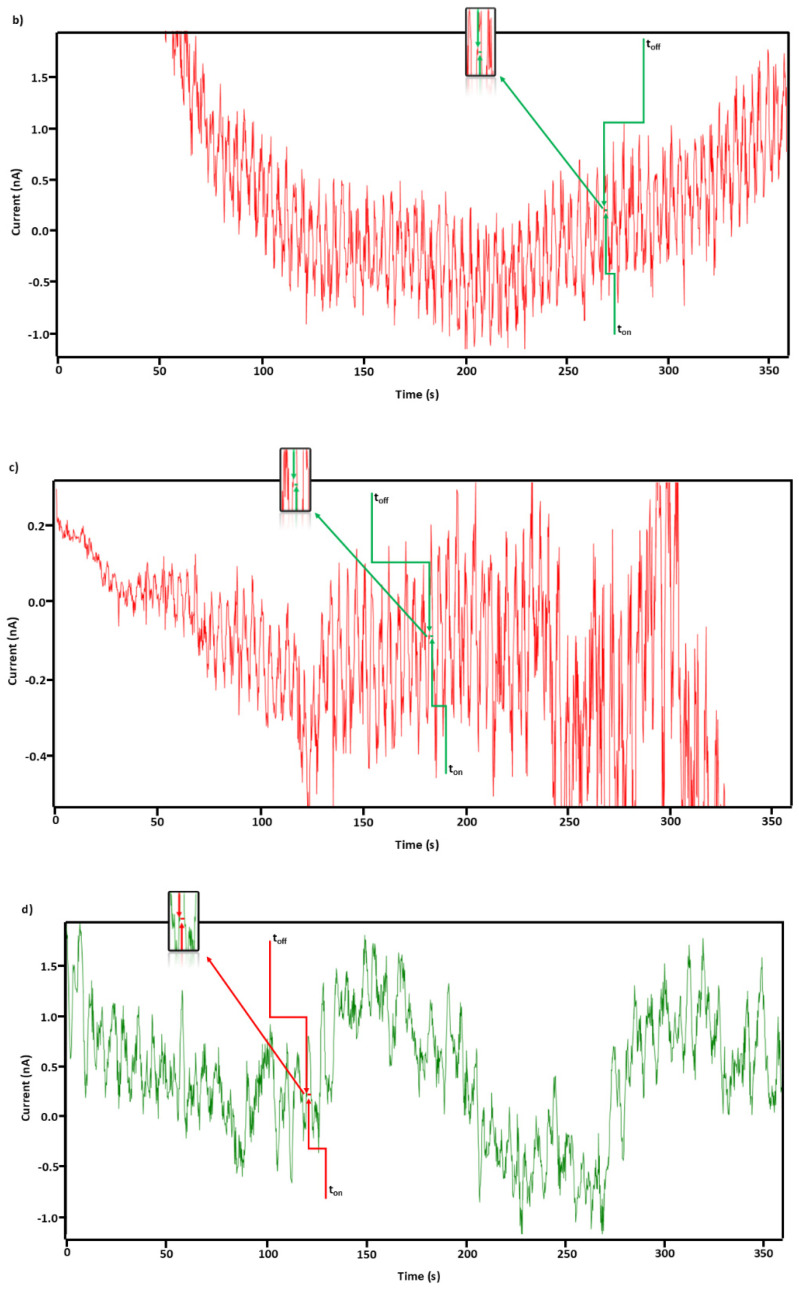
Diagrams obtained for the determination of vitamin B6 in (**a**) surface water; (**b**) avocado; (**c**) aqua vitamin; (**d**) pharmaceutical compounds.

A stochastic method procedure was applied for the determination of vitamin B6 in tablets, vitamin water, and surface water samples. Figure 2 shows the diagrams obtained for the analysis of all these types of samples.

In accordance with the procedure described above, after the identification of the signature of vitamin B6 in the diagram (according to its signature of 0.4 s), the t_on_ value was read and introduced into the calibration equation in order to determine the concentration of vitamin B6.

Ten determinations were performed for the determination of vitamin B6 in Aqua vitamin MG (0.21 mg L^−1^); the concentration obtained for vitamin B6 was 0.20 ± 0.08 mg L^−1^, representing 0.95% recovery from the concentration written on the bottle of vitamin water.

Ten determinations were also performed for the determination of vitamin B6 from avocado. The amount of vitamin B6 determined in avocado was 5.98 ± 0.10 µg vitamin B6 per g of avocado. The recovery of vitamin B6 in avocado was done by addition of 6 mg vitamin B6 to 1 g of avocado; the amount recovered was 5.73 ± 0.12 mg vitamin B6, with a recovery of 95.50% from the added amount.

For all of these samples, a single sensor strip was successfully employed, and no significant changes in the t_off_ and t_on_ values were observed.

The results obtained for the determination of vitamin B6 in pharmaceutical tablets are shown in Table 4.

High recoveries of vitamin B6 in the two types of tablets were recorded versus the amount found on the bottles containing vitamins.

No vitamin B6 was found in the water sample. Therefore, 5 known additions of vitamin B6 were made to see if it can be reliably recovered in this type of sample. The results are shown in Table 5.

High recoveries were also obtained when vitamin B6 was determined in a surface water sample.

The results obtained for the determination of vitamin B6 from pharmaceuticals, vitamin water, avocado, and surface water proved that the ultrasensitive sensing strip can be reliably used for the determination of vitamin B6 in these samples. While the minipotentiostat can send the results to a mobile device (smartphone, tablet, laptop), as well as to the database responsible for either the process control of the pharmaceutical compounds, or food industries, or to databases for the quality of veggies and surface waters, the fast response of the sensing strip can be reliably used for quality control of these samples.

## 4. Conclusions

The ultrasensitive sensing strip displayed the lowest limit of determination compared with those of other methods used for the determination of vitamin B6. Determination of vitamin B6 in different samples like pharmaceutical samples, water vitamins, avocado, and surface water was done with high recoveries and lower relative standard deviations. The sensing strips are stable for at least 30 days when used daily for the determination of vitamin B6. The main features of the sensing strip are for the quality control of pharmaceutical compounds containing vitamin B6, of water vitamins, of vegetables like avocado, as well as of surface water samples.

## Figures and Tables

**Table 1:** Response characteristics of the sensing strip used for determination of vitamin B6.

Linear Concentration Range (g mL^−1^)	Equation of Calibration and Correlation Coefficient (r) *	Signature (s)	Sensitivity (s^−1^ g^−1^ mL)	Limit of Determination (g mL ^−1^)
1.69 × 10^−15^–1.69 × 10^−5^	1/t_on_ = 0.52 + 6.96 × 10^4^ × C_vitamin B6_ r = 0.9991	0.4	6.96 × 10^4^	1.69 × 10^−15^
1.69 × 10^−5^–1.69 × 10^−3^	1/t_on_ = 0.49 + 3.65 × 10^2^ × C_vitamin B6_ r = 0.9999		3.65 × 10^2^	

* <C_vitamin B6_> = g mL^−1^; <t_on_> = s.

**Table 2:** Comparison between the limits of determination obtained using the latest methods used for the determination of vitamin B6, and the limit of determination obtained using the proposed sensing strip.

Method/Tool	Limit of Determination g mL^−1^	Reference
HPLC/MS	1.00 × 10^−11^	[5]
Fluorescence	5.00 × 10^−5^	[7]
Electroanalysis/Ag nanoparticle-polypyrrole nanocomposite/glassy carbon electrode	2.54 × 10^−6^	[11]
Electroanalysis/carbon based sensors	6.77 × 10^−10^	[10]
Electroanalysis/pencil graphite electrode	1.69 × 10^−8^	[13]
This work	1.69 × 10^−15^	-

**Table 3:** The selectivity of the proposed stochastic sensing strip.

Substance	Signature, s
Vitamin B6	0.4 ± 0.1
Mg^2+^	0.1 ± 0.1
Vitamin B1	1.4 ± 0.1
Vitamin B2	1.0 ± 0.1
Vitamin B3	2.2 ± 0.1
Vitamin B5	1.2 ± 0.1
Vitamin B7	1.8 ± 0.2
Vitamin B12	3.2 ± 0.2
Vitamin C	2.6 ± 0.1
Vitamin E	3.6 ± 0.1
Amoxicillin	2.0 ± 0.1
Flurbiprofen	3.0 ± 0.1

**Table 4:** Determination of vitamin B6 in pharmaceutical compounds.

Pharmaceutical Compounds	Mg Vitamin B6 Per Tablet	%, Recovery of Vitamin B6
Complex of vitamins (1.4 mg vitamin B6 per tablet)	1.38	98.57
	1.38	98.57
	1.41	100.71
	1.42	101.43
	1.35	96.43
	1.37	97.86
	1.41	100.71
	1.38	98.57
	1.39	99.39
	1.38	98.57
Vitamin B6 (5 mg vitamin B6 per tablet)	4.90	98.00
	4.85	97.00
	4.86	97.20
	4.86	97.20
	4.87	97.40
	5.01	100.20
	4.90	98.00
	4.96	99.20
	4.93	98.60
	4.95	99.00

**Table 5:** Recovery of vitamin B6 in surface water samples.

Theoretical Concentration of Vitamin B6	Recovered Concentration of Vitamin B6	%, Recovered Vitamin B6
3.20 × 10^−12^	3.15 × 10^−12^	98.44
1.80 × 10^−14^	1.75 × 10^−14^	97.22
5.70 × 10^−10^	5.64 × 10^−10^	98.95
3.20 × 10^−7^	3.19 × 10^−7^	99.69
2.50 × 10^−4^	2.48 × 10^−4^	99.20

## Data Availability

Data will be released upon reasonable request. The data presented are the result of analysing few dozen of diagrams, when hundreds of data were analysed, and processed.

## Supplementary Materials

The following supporting information can be downloaded at: https://www.mdpi.com/article/10.3390/biology15141098/s1, Figure S1: Stability of sensing strip in time. Figure S2: Diagrams recorded for the solution containing vitamin B6 in concentrations of: (a) 1 × 10^−3^ g mL^−1^, (b) 1 × 10^−5^ g mL^−1^, (c) 1 × 10^−10^ g mL^−1^, (d) 1 × 10^−15^ g mL^−1^, and for the (e) buffer solution. And formulas used for statistical calculations.
