# Supplementary material for: Fast Ultrasensitive Sensing Strip for the Electrochemical Determination of Vitamin B6
Source: Biology (Basel). 2026 Jul 8;15(14):1098. doi: 10.3390/biology15141098 (PMC13406081; doi:10.3390/biology15141098)
Supplement: Supplementary file 1 [file biology-15-01098-s001.zip › biology-4373240-supplementary.pdf]

Fast Ultrasensitive Sensing Strip for the Electrochemical Determination of Vitamin B6

SUPPLEMENTARY MATERIAL

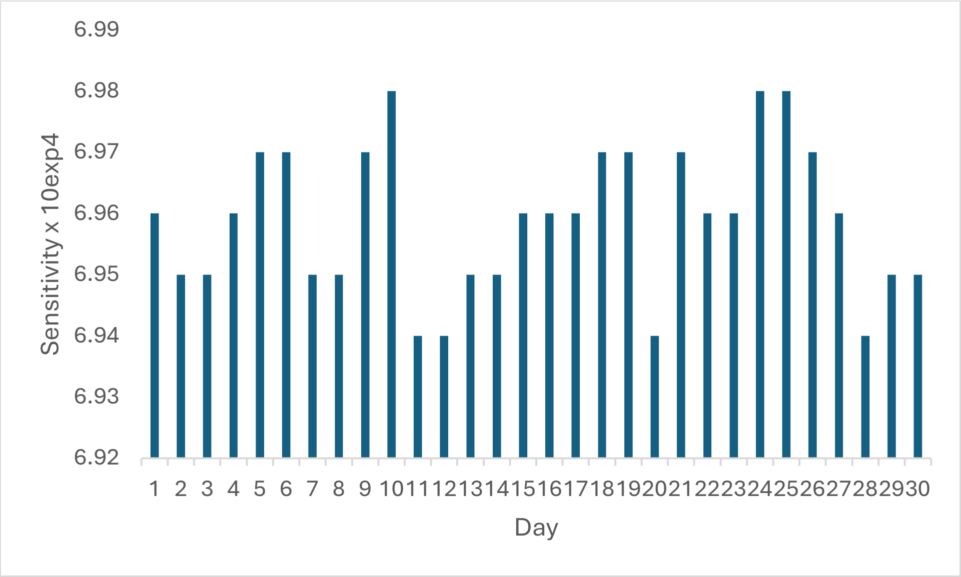

Figure S1 a Stability of sensing strip in time, when calibration on the wider concentration range.

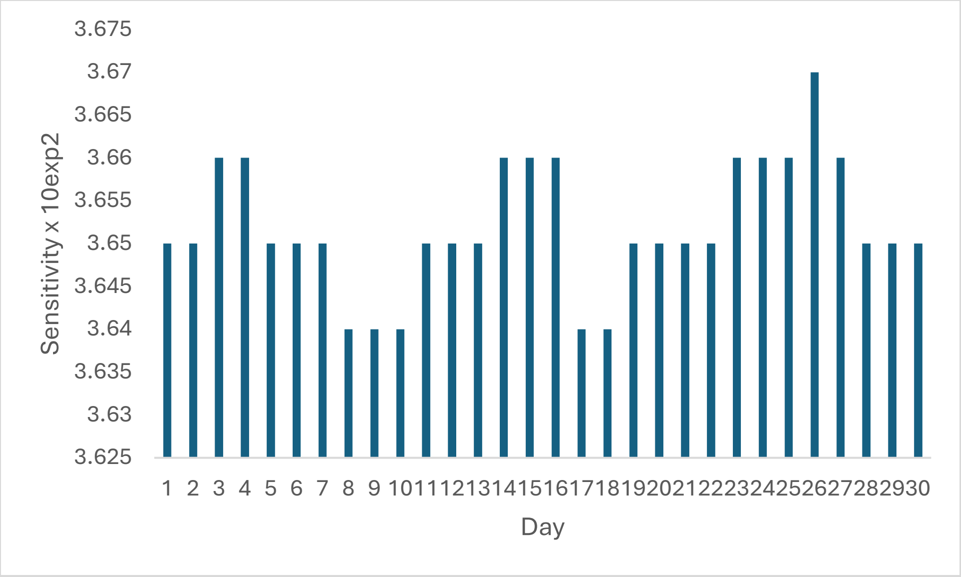

Figure S1 b Stability of sensing strip in time, when calibration on the smallest concentration range.

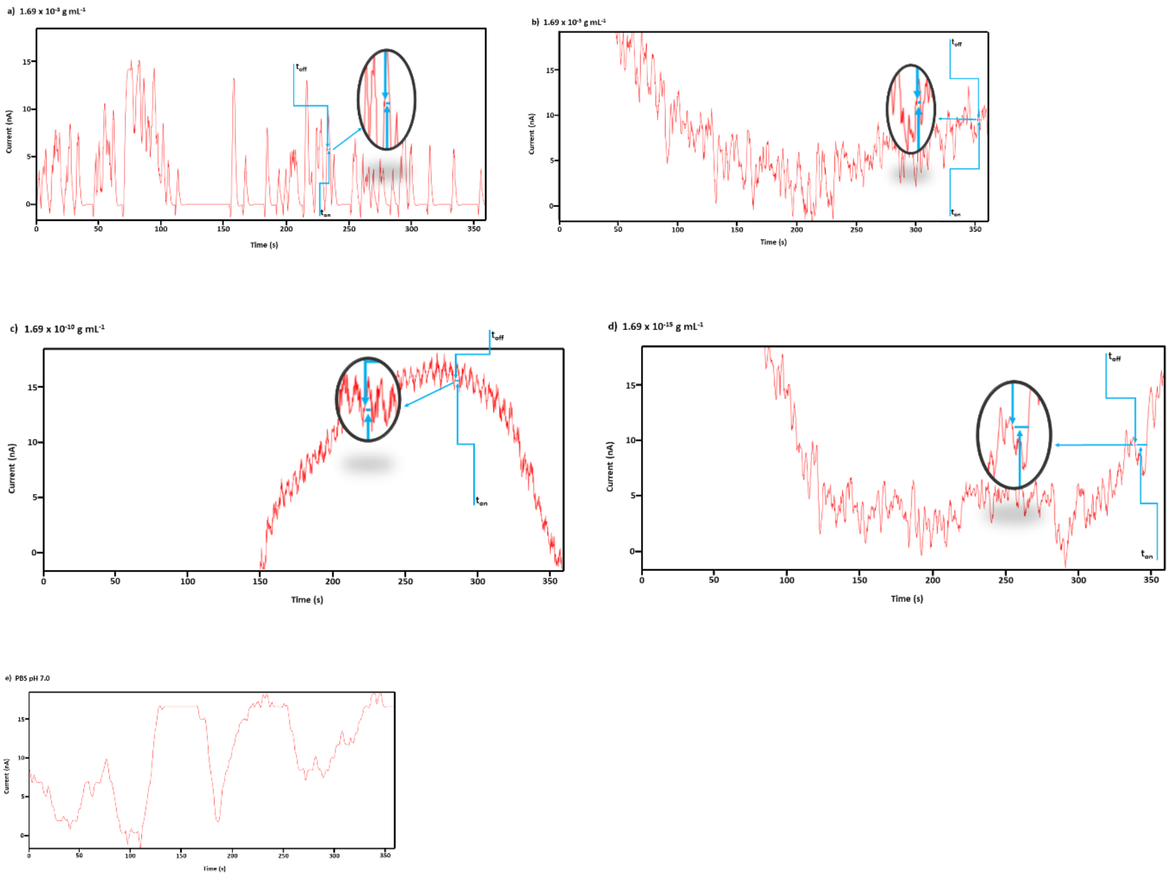

Figure S2 Diagrams recorded for the solution containing vitamin B6 in concentrations of: (a) 1× 10^{-3} g mL^{-1}, (b) 1× 10^{-5} g mL^{-1}, (c) 1× 10^{-10} g mL^{-1}, (d) 1× 10^{-15} g mL^{-1}, and for the (e) buffer solution.

Calculation formulas used by Vassar Stats website for statistical computation (http://vassarstats.net) for a, b, and r parameters of the equation of calibration:

$1/t_{on} = a + b \times C_{vitamin\ B6}$

$$a = \frac{\sum {}^{1}/_{t_{on}} - b \sum C_{vit\ B6}}{3}$$

$$b = \frac{3\sum C_{vit\ B6}\, {}^{1}/_{t_{on}} - \sum C_{vit\ B6} \sum {}^{1}/_{t_{on}}}{3\sum {C_{vit\ B6}}^{2} - (\sum C_{vit\ B6})^{2}}$$

$$r = \frac{3\sum C_{vit\ B6}\, {}^{1}/_{t_{on}} - \sum C_{vit\ B6} \sum {}^{1}/_{t_{on}}}{\sqrt{\left(3\sum {C_{vit\ B6}}^{2} - (\sum C_{vit\ B6})^{2}\right)}\left(3\sum {{}^{1}/_{t_{on}}}^{2} - (\sum {}^{1}/_{t_{on}})^{2}\right)}$$

$1/t_{on}$ values correspond to the concentration of vitamin B6 from the standard solution.
